# Effect of Ultrasonic Treatment on Taste and Flavor Quality of Japonica Rice

**DOI:** 10.3390/foods14091627

**Published:** 2025-05-04

**Authors:** Kaiqing Lian, Lina Guan, Min Zhang, Guodong Ye, Sixuan Li

**Affiliations:** 1 Beijing Engineering and Technology Research Center of Food Additives, Beijing Technology and Business University, Beijing 100048, China; 2 Beijing Advanced Innovation Center for Food Nutrition and Human Health, Beijing Technology and Business University, Beijing 100048, China; 3 School of Food and Health, Beijing Technology and Business University, No. 11 Fucheng Road, Haidian District, Beijing 100048, China

**Keywords:** ultrasound treatment, Japonica rice, resistant starch, rice taste, aroma compounds

## Abstract

The aim of this study was to investigate the effect of ultrasound treatment on the texture and flavor quality of Japonica rice to provide a basis for the development of a staple food product for the treatment of diabetes mellitus. The texture and flavor qualities of cooked rice were analyzed using a texture analyzer, rapid viscosity analyzer (RVA), and gas chromatography–mass spectrometry (GC-MS). The results showed that with increased ultrasound treatment time, the hardness and chewiness of the cooked rice gradually increased, while adhesion decreased. Additionally, the ultrasound treatment reduced various viscosity parameters of rice during the pasting process, inhibiting paste expansion and regrowth. Key aroma compounds influencing the aroma of cooked rice before and after ultrasound treatment included hexanal, heptanal, 2-pentylfuran, octanal, nonanal, trans-2-octenal, decanal, undecanal, trans-2-nonanal, trans-2-dodecenal, trans-2-decenal, trans-2,4-decadienal, 2-pentadecanone, and indole. The odor activity value (OAV) of these compounds increased significantly and were greater than one after ultrasound treatment. These compounds play a role in composing the unique aroma of cooked rice and contribute to sweet, floral, and nutty aromas. In conclusion, ultrasound treatment can be used to increase the content of resistant starch in cooked rice and has a positive effect on the flavor quality of cooked rice.

## 1. Introduction

There is a growing interest in enhancing the health benefits of starchy foods by increasing their resistant starch content [1]. Several methods have been developed to promote the synthesis of resistant starch. The five types of resistant starch have been classified, with the starch–lipid complex (RS5) being one of the most notable [2]. Starch–lipid complexes have been developed to increase the amount of resistant starch in starchy foods, which have attracted significant attention in recent years. Because these complexes are fermented by microorganisms in the large intestine rather than digested in the small intestine, RS5 contributes to postprandial glucose regulation [3]. The ability of rectilinear starch in forming complexes with lipids is mainly attributed to its transformation into a helical structure allowing lipid molecules to interact with the starch core [4]. Direct-chain starch–lipid complexes primarily form through the interaction between hydrogen bonds and hydrophobic forces [4]. The formation of V-helix complexes can modify starch characteristics, such as limiting the starch retrogradation, delaying the aging of straight-chain starch. This modification prevents the recrystallization of branched-chain starch, reduces starch swelling forces, and increases starch resistance to enzymatic hydrolysis [5].

Many chemical, physical, and enzymatic techniques have been employed to accelerate the formation of V-complexes [3,4,5,6]. Recent studies have shown that ultrasound treatment can also facilitate the integration of lipids into starch molecules [4], potentially promoting the formation of V-type complexes. Ultrasound treatment is a cost-effective, efficient, and simple method that can dissolve swollen starch, release linear amylose, enhance lipid dispersibility in gelatinized starch, and facilitate the complexation process between amylose and lipids. These effects can influence the physicochemical and functional aspects of starch [7,8,9]. Studies have shown that the generation of single helix complexes through ultrasound treatment significantly enhances the release of additional linear chains from swollen starch granules and improves lipid dispersion in samples [10]. Additionally, ultrasound treatment facilitates the breakage of branched starch branching points by disrupting the C–O–C bond in the α-1,6 glycosidic bond, leading to an increase in the number of linear chains [3]. However, there has been limited research on the formation of the starch–lipid complex in cooked rice using ultrasound treatment and its effect on cooked rice quality.

Given the increasing prevalence of diabetes, there is a growing demand for staple foods with a low glycemic index. Meanwhile, Japonica rice is widely grown and consumed in China compared to other types of rice. Japonica rice has a soft, delicate texture and is somewhat sticky, which is more for cooking rice. Nutritionally, Japonica rice has a higher fat content than other varieties, which is favorable for the production of starch–lipid complexes. Thus, this study investigates the effect of ultrasound treatment on the formation of starch–lipid complexes and their impact on the quality of rice made from five types of Japonica rice in China. These five types of Japonica rice have more cultivation area and consumption level in China, and they are widely popular. They also have different ratios of amylose and amylopectin. Through this study, rice with a low glycemic index, effective maintenance of postprandial blood glucose stability, and good taste quality can be obtained. It can expand the choice of staple food for diabetic patients and satisfy the desire of special populations for a normal diet and the demand for high-quality and healthy staple food.

## 2. Materials and Methods

### 2.1. Reagents

Solid-phase microextraction (SPME) fibers coated with DVB/CAR/PDMS (divinylbenzene/carboxy/polydimethylsiloxane, 2 cm, 50/30 μm) and 20 mL of headspace vials were purchased from the company Sigma-Aldrich SUPELCO, Shanghai, China. A mixture of n-alkane (C7–C30) was obtained from Sigma-Aldrich. The internal standard 2-methyl-3-heptanone was supplied by the company Beijing Baiodi Biotechnology Co., Beijing, China.

### 2.2. Sample Collection

Five varieties of rice, namely Long Japonica 3013 (LJ3013), Ji Japonica 830 (JJ830), Ji Japonica 816 (JJ816), Ji Japonica 305 (JJ305), and Long Japonica 66 (LJ66), were obtained from the breeding institutes. The five Japonica rice varieties, widely grown in China, are suitable for cooked rice preparation (Rice Research Institute of the Academy of Agricultural Sciences). The five rice were taken at physiological maturity and dried to a moisture content ranging from 12 to 14%.

### 2.3. Instruments and Equipment

The instrument and equipment used in this study included an experimental monopoly grain machine (THU35C, Satake Machinery (Suzhou) Co., Ltd., Suzhou, China), experimental rice mill (TM05C, Satake Machinery (Suzhou) Co. Ltd., Suzhou, China), freeze dryer (LGJ-10 Multi Manifold Ordinary Type, Beijing Tianlin Hengtai Science & Technology Co., Ltd., Beijing, China), texture meter (TMS-Pilot, Food Technology, Inc., Sterling, VA, USA), rapid viscosity analyzer (RVA 4500, Perten Instruments, Inc., Stockholm, Sweden), electronic nose (PEN3, AIRSENSE Analytics, Inc., Land Mecklenburg, Germany), SPME fully automated sampling system (PALRSI, Guangzhou Zhi Da Laboratory Science and Technology Co., Ltd., Guangzhou, China), and gas chromatography–mass spectrometer GC-MS (D7890-5977B, Agilent Technologies, Inc., Santa Clara, CA, USA).

### 2.4. Experimental Methodology

#### 2.4.1. Sample Preparation

Rice Preparation: The rice was first dehulled using a paddy dehuller. Then, it was milled using a rice milling machine, with the milling time determined using the mass of rice bran as 10% of the mass of brown rice as the standard. The milled rice conformed to the standard of first-grade commercial rice as specified in GB/T 1354-2018 [11]. Afterward, the rice samples were sealed in bags and kept at 4 °C for subsequent analysis.

**Figure 1:**
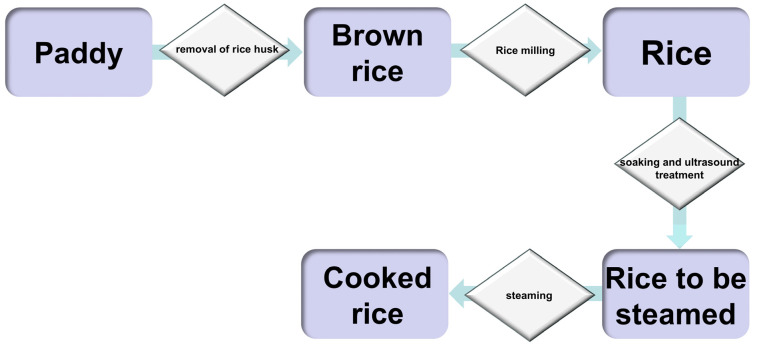
Flow chart of rice cooking and ultrasound treatment.

A small amount of cooked rice sample was prepared following GB/T 15682-2008 [12]. The rice samples were weighed and placed in a closed aluminum box with a lid. Then, an appropriate amount of distilled water was added and washed three times using the water-to-rice mass ratio of 1.5:1. Afterward, distilled water was added to the samples and placed in the aluminum box and treated at 25 °C with ultrasound frequencies of 40 kHz and ultrasound power of 100, 200, and 300 W for 0, 10, 20, and 30 min. After 30 min of immersion, the samples were cage-cooked for 30 min and then simmered for 10 min [13] (Figure 1).

#### 2.4.2. Resistant Starch Content

The AOAC (Association of Official Analytical Chemists testing resistant starch) method was used to determine the content of resistant starch in cooked rice.

#### 2.4.3. Methods of Textural Characterization

The total texture analysis (TPA) mode was used following the slightly modified method of Liu et al. [14]. First, 8 g of cooked rice to be tested was placed into the pressing mold and then pressed for 30 s to prepare rice cakes. The parameters were set as follows: P/36R probe, trigger force of 0.05 N, speed testing of 60 mm/min, and compression ratio of 50%.

#### 2.4.4. Determination of Pasting Characteristics

The pasting characteristics of Japonica rice were determined at different ultrasound treatment times using a rapid viscosity analyzer following the slightly modified method of Han et al. [15]. A sample of 3.00 g of rice flour (at 14% moisture content) was placed into a test jar, and 25 mL of distilled water was added. The mixture was agitated at 960 r/min for 10 s and then at 160 r/min.

#### 2.4.5. Electronic Nose Analysis

Volatile compounds in different varieties of cooked rice were analyzed using a portable electronic nose following the slight modifications of the method of Asimi et al. [16]. A 5 g cooked rice sample was placed in a specialized headspace container and sealed with a double layer of cling film. The sensor parameters were set with an analysis time of 60 s and an injection flow rate of 300 mL/min. Each sample was separately analyzed five times.

#### 2.4.6. Determination of Volatile Compounds Based on SPME-GC-MS

##### Extraction Methods for Volatile Compounds

After the cooked rice was evenly stirred, 5 g of the sample was transferred into a 20 mL SPME vial. Then, 1 μL of 2-methyl-3-heptanone (0.816 μg/μL) was added to the vial as an internal standard and sealed. The samples were equilibrated in a self-heating incubator at 80 °C for 15 min with an oscillation speed of 450 rpm, while the SPME fibers were pretreated by heating them to 250 °C for 5 min. Then, SPME fibers were subjected to headspace, and volatiles were absorbed at 80 °C for 40 min. After the extraction, the fibers were placed into the injection port of a gas chromatograph (250 °C) and desorbed for 5 min.

##### GC-MS Analytical Methods

Volatile chemicals were examined using a mass spectrometer fitted with a sniffer port. The volatiles were separated using a DB-WAX column (30 m × 0.25 mm, 0.25 μm, J&W Scientific, Inc., Agilent Technologies, Inc., Santa Clara, CA, USA). High-purity helium (99.999%) was used at carrier gas at a constant flow rate of 1.2 mL/min, and the injection was performed in non-split flow mode under the following GC conditions: The temperature was maintained at 40 °C for 3 min. Afterward, the temperature was increased to 200 °C at 5 °C/min and further increased to 230 °C at 5 °C/min for 3 min. The mass spectrum was obtained over the scanning range of *m*/*z* 55–500 at a scanning interval of 0.5 s [17]. The retention index of each compound was calculated using n-alkanes (C7–C30) under the same analytical parameters as the samples (split ratio: 30:1). The solvent delay time was set to 6 min. Preliminary identification of the volatile components was achieved by comparing their actual mass spectral data with the reported mass spectral data and RI from the authentic NIST 14 mass spectral library. N-alkanes that were injected under the same chromatographic conditions used to calculate the RI. The internal standard semi-quantitative method was used for quantitative analyses. The content of volatile compounds in the cooked rice samples was calculated using the content of 2-methyl-3-heptanone as follows:

*C* (μg/kg) = *PC*/*Pis* × *Cis* × 1000/*m*_0_

where *C* is the content of volatile compounds, *Cis* is the content of 2-methyl-3-heptanone, *PC* is the peak area of volatile compounds, *Pis* is the peak area of 2-methyl-3-heptanone, and *m*_0_ is the weight of the cooked rice sample.

In addition, the relative odor activity value (rOAV) of each compound was calculated to assess the contribution of each ingredient to the aroma of cooked rice. The corresponding aroma thresholds recorded in the literature and the concentration of individual chemicals in the test samples were used to calculate aroma intensity.

### 2.5. Statistical Analysis

All treatments in this study were repeated three or more times to ensure the accuracy of the study. Data processing was conducted using Microsoft Excel 2019. SPSS 20.0 was used to perform the analysis of variance and significance (ANOVA). The software program Origin (version 2022, MicroCalInc., Northampton, MA, USA) and the software package GraphPadPrism 8 were used to perform the graphing. The GC-MS data were analyzed by searching NIST14 spectral libraries and the corresponding literature, and peak area normalization was used to calculate the relative content of each compound. The volatile compound data were analyzed using a heat map using Hiplot 3.0 (https://hiplot.com.cn/).

## 3. Results

### 3.1. Changes in Resistant Starch Content

**Figure 2:**
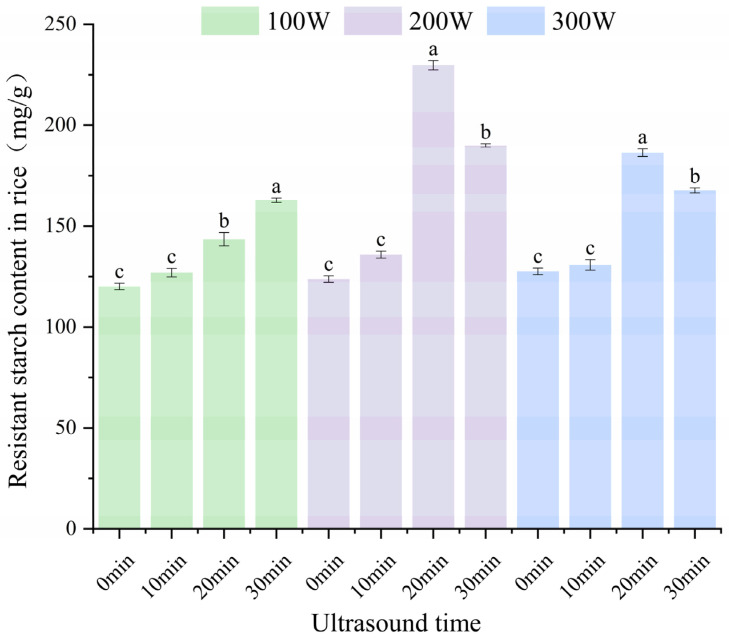
Effect of ultrasound treatment on amount of resistant starch in cooked rice JJ830. Different letters under the same parameter indicate the significant differences (*p* < 0.05).

The effect of ultrasound treatment on resistant starch content in cooked rice JJ830 is shown in Figure 2. The effect of ultrasound treatment on resistant starch content in cooked rice was not significant under low ultrasound treatment intensity (100 W, 10 min). However, there was a significant increase in resistant starch content at 200 and 300 W for 20 min of treatment. The resistant starch concentration at 200 W was higher than that at 300 W. The resistant starch content increased from 123.75 mg/g to 229.76 mg/g, reflecting an 85.66% increase. The above phenomenon is attributed to the fact that ultrasound treatment with medium power density and longer duration may be more favorable for the formation of complexes, whereas vigorous ultrasound treatment (300 W, 30 min) may disrupt the amylose chains and produce short chains that are too short to participate in the production of single helix complexes, which reduces the number of complexes [10]. At the same time the powerful shock waves and mechanical forces of high-intensity ultrasound treatment can disrupt the structure of the already formed starch–lipid complexes. Based on these results, the subsequent experiment was conducted using 200 W ultrasound power.

### 3.2. Properties of Textures

**Figure 3:**
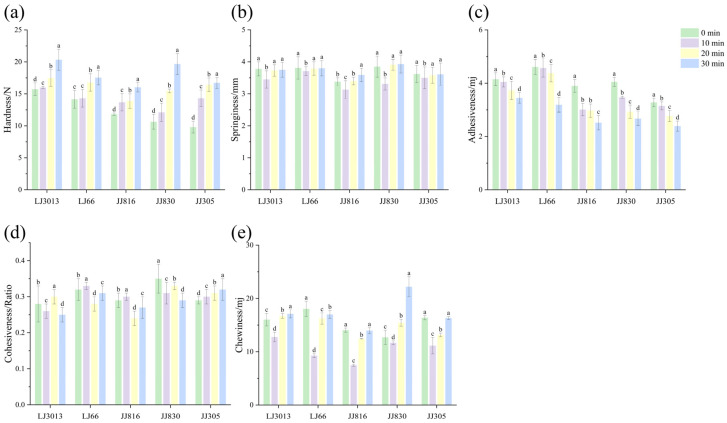
The effect of ultrasound treatment on cooked Japonica rice texture and quality. Effect of ultrasound treatment on cooked rice hardness (**a**); Effect of ultrasound treatment on cooked rice springiness (**b**); Effect of ultrasound treatment on cooked rice adhesiveness (**c**); Effect of ultrasound treatment on cooked rice cohesiveness (**d**); Effect of ultrasound treatment on cooked rice chewiness (**e**). Different letters under the same parameter indicate the significant differences (*p* < 0.05).

Figure 3 illustrates the effect of ultrasound treatment time on the textural characteristics of cooked Japonica rice. The hardness of the cooked rice significantly increased with increasing ultrasound treatment time (*p* < 0.05). This increase was attributed to the mechanical force of the ultrasound, cavitation effect, and strong shock wave, which can break down the swollen starch granules and partially depolymerize straight-chain starch. The release of linear straight-chain starch and the improvement of lipid dispersion facilitated the complexation reaction between straight-chain starch and lipid to form a complex [18], increasing the hardness. After an ultrasound treatment time of 30 min, JJ830 hardness increased by 84.95%. In contrast, LJ66 hardness increased by only 23.94%, indicating that the hardness of JJ830 increased the most with increasing ultrasound time. The difference in hardness between the varieties became significant due to the variability in the body composition of the cooked rice. For example, JJ830 exhibited the highest hardness owing to its high-branched amylose content (75.65%) and the lowest ratio of amylose and amylopectin (0.15), while LJ66 exhibited the least hardness due to its low-branched amylose content (68.89%) and the high ratio of amylose and amylopectin (0.27). Cooked rice adhesion decreased with increasing ultrasound treatment time because ultrasound treatment broke the C–O–C bond of the α-1,6 glycosidic bond, which contributed to the branching point of the branched starch [18]. As the concentration of branched starch decreased, the adhesion progressively decreased. When resistant starch and straight-chain starch contents increased, cooked rice chewability increased, with no significant effect on elasticity and cohesion. This observation is in line with that of earlier research [19]. In summary, high-branched amylose content and low rectilinear ratio varieties facilitated resistant starch production after ultrasound treatment. At 20 min of ultrasound treatment, cooked rice exhibited moderate hardness and viscosity, chewy, minimal gritty perception, and good textural quality.

### 3.3. Pasting Characteristics

The results showed that ultrasound treatment reduced various viscosities of cooked rice samples during the pasting process (Table 1). As ultrasound time increased, the peak viscosity, trough viscosity, and final viscosity of the sample gradually decreased, reaching a minimum after 30 min treatment. Ultrasound treatment breaks branched starch and releases more linear starch. When the content of branched starch in the sample is higher, it is easier to form a paste, whereas when the content of linear starch is higher, it is more difficult to form a paste, so the viscosity decreases. The viscosity reduction in different varieties after ultrasound treatment varies. This phenomenon may be due to the different varieties of the ratio of amylose and amylopectin. The higher the content of branched starch, the more affected by ultrasound treatment, the more linear starch formed after ultrasound treatment [4]. At the same time, the regeneration value also decreased.

This observation indicated that ultrasound treatment enhanced branched-chain starch decomposition and facilitated straight-chain starch depolymerization. The depolymerized straight-chain starch formed complexes with lipids, indicating that lipids were incorporated into the starch helices to form resistant starch. This complexation reduced the likelihood of lipid leaching from the helices, preventing the penetration of water into the starch grains. This finding showed that ultrasound treatment increased the resistant starch content in cooked rice and inhibited its pasty expansion and starch regeneration.

### 3.4. Analysis of Electronic Noses

**Figure 4:**
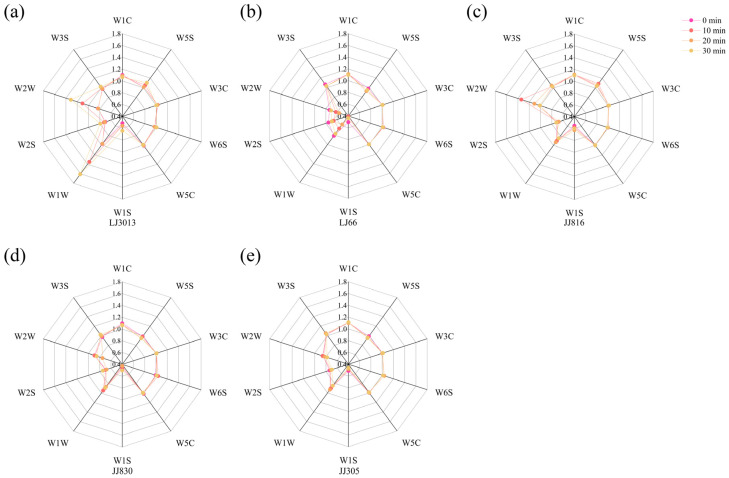
Electronic nose response radar images of five types of cooked Japonica rice under different ultrasound treatments. Radar response map of the electronic nose of LJ3013 under different ultrasound treatments (**a**); Radar response map of the electronic nose of LJ66 under different ultrasound treatments (**b**); Radar response map of the electronic nose of JJ816 under different ultrasound treatments (**c**); Radar response map of the electronic nose of JJ830 under different ultrasound treatments (**d**); Radar response map of the electronic nose of JJ305 under different ultrasound treatments (**e**).

The response values recorded by the electronic nose after 60 s were selected for data analysis to ensure detection accuracy and experimental consistency [20]. Five distinct varieties of cooked Japonica rice, subjected to a range of ultrasound treatments, are represented in the radar plots shown in Figure 4. Among the sensors used, W1C, W3C, W6S, W5C, and W3S exhibited relatively weak signal strengths, with their response values converging at a specific point. In contrast, the W1W and W2W sensors recorded the highest signal intensities, while W1S, W2S, and W5S also exhibited elevated signal levels, indicating that ultrasound significantly influenced their response values. These findings showed that aromatic compounds, including benzene, ammonia, hydride, and alkanes, slightly impacted the ultrasound treatment of cooked Japonica rice. Additionally, variations in aromatic compounds, such as sulfur-containing substances, alcohols, aldehydes, ketones, methyl, and nitrogen oxides, were observed in cooked rice samples treated at varying ultrasound times (Appendix A Table A1). The response values for sulfur-containing substances in LJ3013 and LJ66, aldehydes in LJ66 and JJ830, and aromatic components in LJ3013, LJ66, and LJ816 exhibited significant variations.

### 3.5. GC-MS Data Analysis

#### 3.5.1. Composition of Aroma Compounds

**Figure 5:**
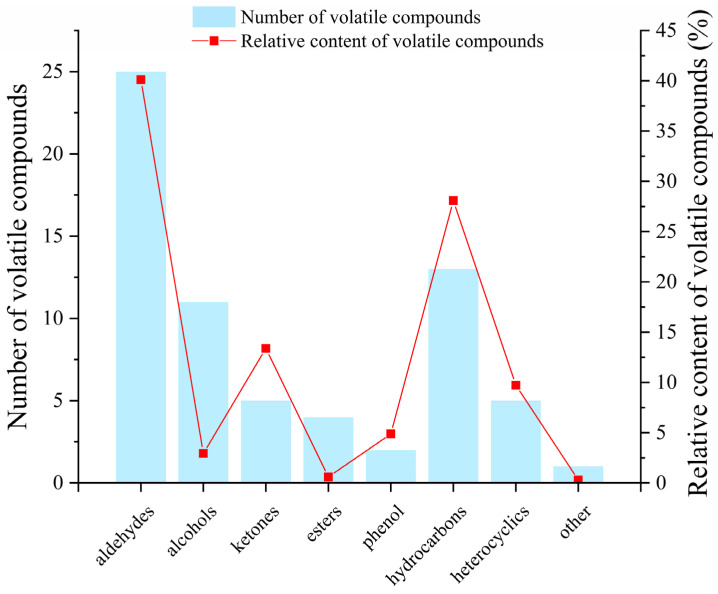
The relative content and quantity of different volatile chemicals in cooked Japonica rice following ultrasound treatment.

A total of 65 volatile compounds in cooked rice were identified in this study. The relative contents of each compound were quantitatively calculated by the peak area normalization method. The quantities and relative contents of various volatile compounds in cooked rice are shown in Figure 5. Out of the 65 volatile compounds detected, 23 (40.1%) were aldehydes, 11 (2.94%) were alcohols, 5 (13.38%) were ketones, 3 (0.6%) were esters, 2 (4.88%) were phenols, 13 (28.08%) were hydrocarbons, 7 (9.72%) were heterocyclic compounds, and 1 (0.3%) was other compounds. The main volatile compounds in cooked rice were aldehydes, heterocyclic compounds, and hydrocarbons, which aligns with the findings of previous studies [21].

**Figure 6:**
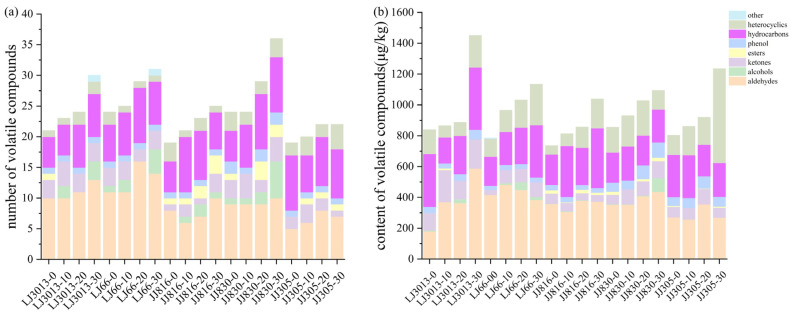
Quantity (**a**) and content (**b**) of various volatile substances in cooked Japonica rice after different ultrasound treatments.

The changes in the number and content of various types of volatile compounds in the five types of cooked Japonica rice after ultrasound treatment are shown in Figure 6. The formation of cracks on the surface of cooked rice grains caused by ultrasound treatment facilitated the penetration of water into the grains during cooking, thereby enhancing the release of volatile compounds [22]. This phenomenon might be attributed to the progressive increase in the types of volatile compounds with increasing ultrasound treatment time. Meanwhile, ultrasound treatment increased lipid dispersion in the sample [10], facilitating lipid oxidation, thereby increasing lipid oxidation products, such as aldehydes and alcohols. The total concentration of volatile compounds gradually increased, mainly due to the increase in the concentration of hydrocarbons and heterocyclic compounds.

#### 3.5.2. Aroma Characteristics

**Figure 7:**
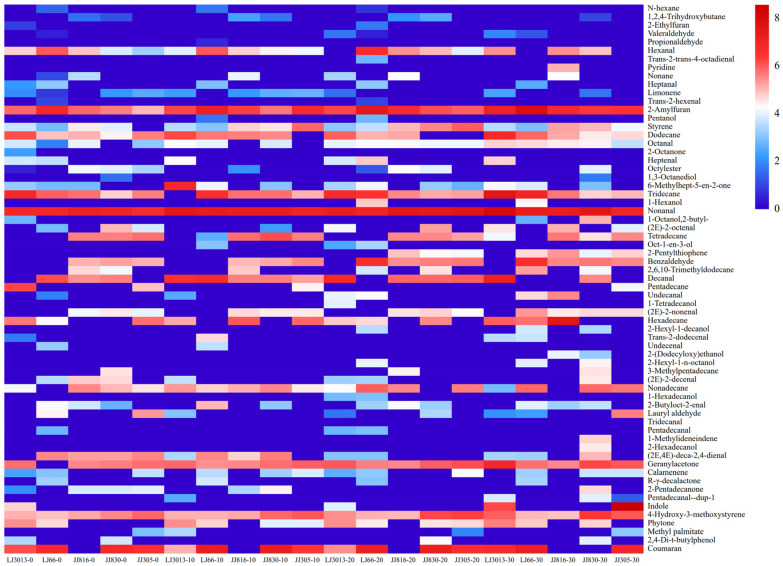
After various ultrasound procedures in different cooked Japonica rice, heat map analysis of volatile compounds was conducted.

To further analyze the differences in volatile compounds between samples, heat maps were plotted for 65 volatile compound contents in five samples of cooked Japonica rice under different ultrasound treatments (Figure 7). The color scale of the heat map was generated using normalized data on the horizontal axis, where blue and red represent minimum and maximum, respectively, clearly differentiating the different levels of content.

Hydrocarbons were the main volatile compounds detected in ultrasound treatment cooked Japonica rice, leading to an increase in the overall concentration of volatile compounds. These hydrocarbons mainly originated from the oxidation and degradation of lipids [13]. Nevertheless, only a small number of alkanes produced distinctive aromas, which had a minimal effect on the overall aroma of cooked Japonica rice. For instance, tridecane, tetradecane, and styrene contributed to sweet aromas, floral aromas, and a blend of sweet and floral aromas, respectively [23]. However, other hydrocarbons slightly influenced the total aroma profile [24]. In addition, trace amounts of heterocyclic compounds were detected in cooked Japonica rice, with their concentrations significantly increasing as ultrasound treatment time was extended. Among the volatile compounds, 2-pentylfuran, the main alkyl furan in cooked rice, was detected in every cooked Japonica rice sample, with its concentration increasing significantly with increased ultrasound treatment time, offering a fruity, nutty, and caramel aroma [23]. Moreover, pyridine and indole were detected in the ultrasound treatment cooked Japonica rice samples, contributing floral notes, while 2,3-dihydrobenzofuran was detected in the cooked rice samples, adding sweetness to the overall aroma [25].

A total of 23 aldehydes were detected in this study, among which nonanal was the highest and was found in all treated samples. The oxidative decomposition of lipids in cooked rice was the primary pathway for the formation of aldehydes. Most of them had high rOAVs and contributed to the aroma of cooked rice, presenting fruity, sweet, and grassy notes. Octanal is a lipid oxidation product of linoleic and oleic acids, whereas decanal is an oxidation product of oleic acid [26]. Aldehydes primarily exhibited fruity, sweet, and grassy aromas. Hexanal and 2-butyl-2-octenal were selected as odor identification markers for cooked rice [27]. The remaining aldehydes, including benzaldehyde and trans-2, 4-decadienal, exhibited a fatty aroma and nutty aromas. These compounds significantly influenced the aroma of cooked rice. The concentration of aldehydes varied after ultrasound treatment. The increase in concentration might be attributed to the ultrasound treatment process that enhanced the dispersion of lipids in the sample and promoted certain oxidative pathways. In contrast, the decrease in concentration was attributed to the binding of lipids and starch, which inhibits their oxidative decomposition process. High concentrations of aliphatic aldehydes, such as hexanal, negatively affected the aroma of cooked rice, resulting in an unpleasant odor [27]. However, the reduction in the concentration of some aldehydes diminished their negative effect on the aroma of cooked rice.

A range of alcohols and ketones were also identified within the volatile compounds extracted from ultrasonically processed cooked Japonica rice. Alcohols, which are secondary metabolites, are formed through the oxidation of unsaturated fatty acids that result from the subsequent breakdown of aldehydes. Ketones are derived from the oxidative degradation of unsaturated fatty acids and the degradation or synthesis of amino acids [28]. At different intensities of ultrasound treatment, the levels of alcohol remained relatively constant. Additionally, ketones were generated through the oxidative breakdown of unsaturated fatty acids and the degradation and synthesis of amino acids [28]. The non-aromatic saturated ketone geranyl acetone was consistently detected in cooked Japonica rice samples under all treatment conditions, contributing a floral aroma. Geranyl acetone positively influenced the aroma of cooked rice [23]. As ultrasound treatment time increased, the concentration of geranyl acetone significantly increased, thereby enhancing the role of ketones in the overall aroma profile of the cooked rice.

In addition to the volatile compounds mentioned above, ultrasound treatment cooked Japonica rice contained esters and phenolic compounds, which contributed to the aroma complexity of cooked Japonica rice. Esters are produced through the esterification of carboxylic acids and alcohols. Phenols are redox products that are decarboxylated by phenolic acids during heating [29]. Phenols, such as 2-methoxy-4-vinylphenol, are mainly associated with smoking. 2-Methoxy-4-vinylphenol, a derivative of guaiacol, contributes to an unpleasant nutty, pungent, and clove-like odor [30]. The main increase in volatiles after ultrasound treatment was mainly in aldehydes and heterocycles compared to traditional cooked rice. Ultrasound treatment is different from the high hydrostatic pressure (HHP), the high hydrostatic pressure had a stabilizing effect on the low-molecular-weight volatiles, resulting in an increase in the alcohol, ketone, ester, and olefin content of the rice, but a decrease in the heterocyclic, alkane, and aromatic content [29]. Ultrasound treatment maintained the alcohol, ketone, and ester contents while increasing the aldehyde and heterocyclic contents. The increase in phenolic concentration caused by ultrasound treatment could have an adverse effect on the aroma of the cooked rice, further requiring validation screening. The concentrations of the remaining phenolic and ester compounds remained unchanged after different ultrasound treatments, which ensured the complexity of the cooked rice aroma after ultrasound treatment.

#### 3.5.3. Analysis of Aroma Compounds

To evaluate the contribution of each volatile compound to the total change in the cooked rice aroma following ultrasonography treatment [31], the rOAV obtained by dividing the concentration of an aroma compound by its odor threshold in air was used in addition to the concentration analysis. Compounds that significantly contribute to the aroma, i.e., compounds with rOAVs ≥ 1 (Table 2), were screened and analyzed [32,33]. Numbers in parentheses below represent the change in rOAVs of the aroma compounds without and after ultrasound treatment.

Although hydrocarbons constituted most of the aroma substances, most of them exhibited high thresholds and contributed less to the aroma of cooked rice. In contrast, aldehydes, heterocyclics, and alcohols exhibited low thresholds and mainly contributed to the cooked rice aroma. Straight-chain alkanes, such as tridecane and tetradecane, exhibited low rOAVs and contributed less to cooked rice aroma. 2-Pentylfuran (5.47–41.46) had a fruity, nutty, and caramelized aroma [29].

Among the detected aldehydes, hexanal (2.83–20.89), octanal (3.30–32.90), nonanal (96.5–254.44), decanal (12.35–47.06), trans-2-octenal (1.23–12.26), trans-2-nonenal (82.58–205.58), and trans, trans-2,4-decadienal (3.09–21.40) had rOAVs greater than 1, indicating their significant contribution to the aroma of cooked rice, with a significant range of variation. In addition, trans-2-decenal (0.51–1.63), undecanal (0.20–3.58), and trans-2-dodecenal (0.31–3.31) had rOAVs ranging from below to above 1. Aldehydes significantly affected the change in cooked rice aroma after ultrasound, and the ultrasound treatment promoted the oxidative decomposition process of aldehydes during rice cooking.

The ROAVs of alcohol were low, and only n-hexanol (3.50–5.08) and 1-octen-3-ol (2.97–6.16) were detected in all the samples, and their rOAVs were greater than 1 but with slight variations, presenting fruity aromas and mushroom aromas.

Geranyl acetone was detected in all samples but with low rOAVs, which slightly affected the aroma of the cooked rice before and after ultrasound treatment. 2-Pentadecanone (0.44–2.89) and indole (0.38–9.48) exhibited rOAVs ranging from below 1 to above 1. Ultrasound treatment affected these aroma compounds during the formation of the aroma of the cooked rice.

Esters and phenols, in addition to the volatile chemicals listed above, were attributed to the complicated aroma of the cooked rice. The low rOAVs of esters and their decreased aroma also contributed less to the aroma of the cooked rice. Phenols are redox products and are decarboxylated by phenolic acids during heating [29]. Phenols, such as 2-methoxy-4-vinylphenol (2.39–7.01), are mainly associated with smoky aromas; it is a derivative of guaiacol and has an unpleasantly nutty, pungent, and clove odor [30]. Slight changes in ester and phenolic rOAVs slightly affected the aroma of the cooked rice after ultrasound treatment.

Hence, the most dominant compounds in the aroma of the cooked rice were hexanal, heptanal, 2-pentylfuran, octanal, nonanal, trans-2-octenal, decanal, undecanal, trans-2-nonanal, trans-2-dodecenal, trans-2-decenal, trans-2,4-decadienal, 2-pentadecanone, and indole before and after ultrasound treatment. The 14 compounds mentioned above were the volatile aroma compounds that significantly influenced the aroma of the cooked rice before and after ultrasound treatment. These compounds showed similar compositions to the key aroma compounds identified in cooked rice in previous studies [21].

### 3.6. Correlation Analysis

**Figure 8:**
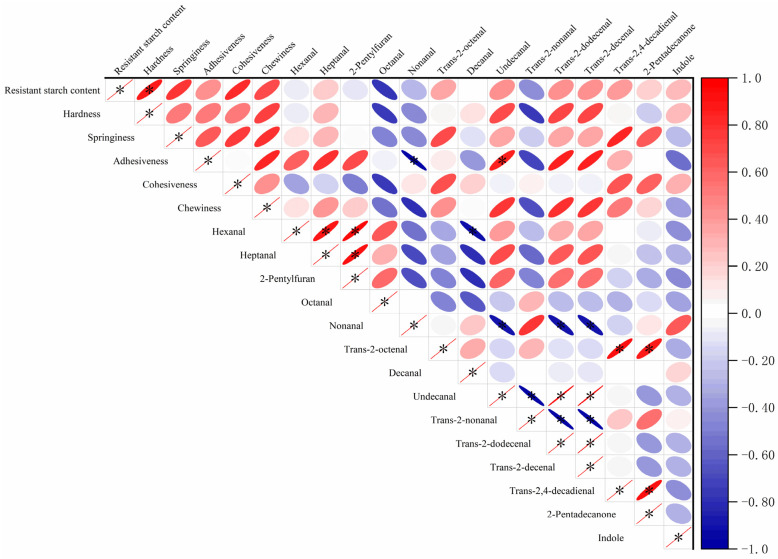
Resistant starch content was analyzed in correlation with aroma compounds and texture parameters.“*” in the figure indicates significant differences (*p* < 0.05).

Finally, Pearson correlation analysis was performed to investigate the relationship among rice resistant starch content, texture parameters, and aroma compounds. The correlation of resistant starch content in the cooked rice with textural parameters and aroma compounds is shown in Figure 8. The hardness of rice was significantly and positively correlated with resistant starch content (*p* < 0.05). The correlations between the textural parameters and aroma compounds of the ultrasound treatment cooked rice were similar to previous studies [34]. Octanal was negatively correlated with the hardness of the cooked rice. Hexanal, heptanal, and 2-pentylfuran showed significant positive correlation (*p* < 0.05) as they are all products of initial lipid oxidation of linoleic acid [35]. Trans-2-nonenal and trans-2,4-dodecenal also showed a significant positive correlation (*p* < 0.05) as they are both products of late lipid oxidation of linoleic acid [35]. Therefore, resistant starch has a correlation with the texture and flavor of ultrasound treatment rice.

## 4. Conclusions

In this study, changes in the quality of five different ultrasound treatment cooked Japonica rice samples were investigated. The results show that ultrasound treatment causes the branched starch in cooked rice to break, releasing more linear starch and unspinning it. It also increases lipid dispersion and facilitates lipid entry into the opened starch helix, resulting in the formation of starch–lipid complexes. The ultrasound treatment mainly affected the hardness, adherence, and chewiness of the cooked rice in terms of textural characteristics. As the ultrasound time increased, the hardness and chewiness gradually increased, whereas the adherence gradually decreased. In addition, the ultrasound treatment reduced the viscosity of cooked rice during the pasting process. As ultrasound treatment time increased, peak viscosity, trough viscosity, final viscosity, and regrowth value were reduced. The concentration of volatile compounds in the five cooked Japonica rice samples increased gradually with extended ultrasound treatment time. This increase was mainly attributed to the increasing levels of hydrocarbons, aldehydes, and heterocyclic compounds. The main volatile components that exhibited significant changes in the cooked rice samples after ultrasound treatment included hexanal, heptanal, 2-pentylfuran, octanal, nonanal, trans-2-octenal, decanal, undecanal, trans-2-nonanal, trans-2-dodecenal, trans-2-decenal, trans-2,4-decadienal, 2-pentadecanone, and indole. Ultrasound treatment has a positive effect on the flavor quality of rice.

Studies on enhancing starch–lipid complexes in cooked rice using ultrasound treatment are limited. As a result, the current findings offer valuable insight into the changes in the quality of cooked rice treated with ultrasound. Future studies will explore the effect of ultrasound treatment on different cooked rice varieties, focusing on variations in their basic components and aroma formation process. The study confirmed the feasibility of ultrasound in industrialized production. This study also confirms that ultrasound treatment can increase the content of resistant starch, but some animal experiments and clinical data are needed to really realize the industrial production of ultrasonicated cooked rice for the purpose of treating those with diabetes.

## Figures and Tables

**Table 1:** Effect of ultrasound treatment time on pasting characteristics.

Sort	Ultrasound Time/min	Peak Viscosity/cP	Trough Viscosity/cP	Final Viscosity/cP	Retrogradation Value/cP
LJ3013	0	1590 ± 13 ^a^	1541 ± 12 ^a^	2333 ± 45 ^a^	743 ± 4 ^a^
	10	1516 ± 13 ^b^	1482 ± 32 ^b^	2207 ± 23 ^b^	691 ± 8 ^b^
	20	1424 ± 28 ^c^	1387 ± 28 ^c^	2075 ± 8 ^c^	651 ± 13 ^c^
	30	1400 ± 18	1372 ± 16 ^c^	2032 ± 13 ^c^	632 ± 8 ^c^
LJ66	0	1499 ± 21 ^a^	1502 ± 23 ^a^	2209 ± 49 ^a^	710 ± 6 ^a^
	10	1402 ± 16 ^b^	1406 ± 5 ^b^	2119 ± 17 ^b^	717 ± 9 ^a^
	20	1388 ± 5 ^b^	1389 ± 0 ^c^	2082 ± 11 ^c^	694 ± 1 ^a^
	30	1315 ± 22 ^c^	1318 ± 27 ^d^	1956 ± 13 ^d^	641 ± 21 ^b^
JJ816	0	1574 ± 9 ^a^	1541 ± 18 ^a^	2396 ± 13 ^a^	822 ± 21 ^a^
	10	1406 ± 13 ^b^	1375 ± 1 ^b^	2130 ± 22 ^b^	724 ± 6 ^b^
	20	1170 ± 3 ^c^	1171 ± 70 ^c^	1827 ± 9 ^c^	657 ± 12 ^c^
	30	868 ± 98 ^d^	870 ± 111 ^d^	1365 ± 98 ^d^	497 ± 9 ^d^
JJ830	0	1769 ± 21 ^a^	1664 ± 49 ^a^	2684 ± 40 ^a^	1016 ± 22 ^a^
	10	1672 ± 7 ^b^	1406 ± 52 ^c^	2527 ± 23 ^b^	915 ± 4 ^b^
	20	1571 ± 0 ^c^	1500 ± 24 ^b^	2457 ± 37 ^c^	886 ± 18 ^c^
	30	1424 ± 41 ^d^	1280 ± 45 ^d^	2440 ± 22 ^c^	855 ± 24 ^d^
JJ305	0	1975 ± 14 ^a^	1980 ± 27 ^a^	2942 ± 22 ^a^	983 ± 15 ^a^
	10	1819 ± 28 ^b^	1818 ± 16 ^b^	2711 ± 13 ^b^	967 ± 1 ^b^
	20	1799 ± 22 ^b^	1804 ± 4 ^b^	2636 ± 19 ^c^	892 ± 18 ^c^
	30	1550 ± 71 ^c^	1550 ± 52 ^c^	2533 ± 62 ^d^	837 ± 34 ^d^

Note: Values are given as mean ± standard deviation from triplicate determinations. Different letters under the same parameter indicate the significant differences (*p* < 0.05).

**Table 2:** Aroma characteristics and thresholds of volatile aroma compounds.

No.	Aroma Compounds	Aroma Characteristics	Odor Threshold (μg/kg)	Before Ultrasound rOAV	After Ultrasound rOAV
1	Pentanal	Almond, malt, pungent	20	<1	<1
2	Hexanal	Grass	5	2.83	20.89
3	Heptanal	Fat, citrus, rancid	6	0.61	1.31
4	Pyridine	Sour, fishy	2000	<1	<1
5	2-Pentylfuran	Beany	5.8	5.47	41.46
6	1-Pentanol	Sweet bread	150.2	<1	<1
7	Styrene	Floral	65	<1	<1
8	Octanal	Citrus	0.8	3.30	32.90
9	(E)-2-Heptenal	Fresh, green	3.75	2.97	7.41
10	6-Methyl-5-heptene-2-one	Citrus	59	<1	<1
11	Tridecane	Sweet	42,000	<1	<1
12	1-Hexanol	Fruity	5.6	3.50	5.08
13	Nonanal	Citrus	1.1	96.5	254.44
14	(E)-2-Octenal	Cucumber	3	1.23	12.26
15	Decanal	Citrus	3	12.35	47.06
16	(E)-2-Nonenal	Cucumber	0.19	82.58	205.58
17	1-Octen-3-ol	Raw mushroom	1.5	2.97	6.16
18	(E)-2-Decenal	Citrus	17	0.51	1.63
19	Undecanal	Sweet, floral	12.5	0.20	3.58
20	(E)-2-Dodecenal	Citrus, waxy	7.3	0.31	3.31
21	Geranyl acetone	Fresh, fruity	186	<1	<1
22	(E,E)-2,4-Decadienal	Citrus	2.3	21.40	3.09
23	n-Hexadecanol	Floral	1100	<1	<1
24	2-Pentadecanone	Celery	7	0.44	3.55
25	Pentadecanal	Waxy	1000	<1	<1
26	2-Methoxy-4-vinylphenol	Smoky	12.02	2.39	7.01
27	Indole	Floral	40	0.38	9.48

Note: The odor threshold is referenced in a book called *Odor Threshold Compilation in Air, Water, and Other Media (Second Edition Expanded and Revised)*. Aroma characteristics accessed from http://flavornet.org/flavornet.html (accessed on 18 February 2025).

## Data Availability

The presented data is contained within the article.

## Abbreviations

The following abbreviations are used in this manuscript:

AOAC	Association of Official Analytical Chemists testing resistant starch

## Appendix A

**Table A1:** The type of substance represented by the sensor.

Sensor Number	Sensor Name	Substance Type	Sensitivity Levels
1	W1C	aromatic	Toluene, 10 ppm
2	W5S	broad range	NO_2_, 1 ppm
3	W3C	aromatic	Benzene, 10 ppm
4	W6S	hydrogen	H_2_, 100 ppb
5	W5C	arom-aliph	Propane, 1 ppb
6	W1S	broad-methane	CH_3_, 100 ppm
7	W1W	sulfur-organic	H_2_S, 1 ppm
8	W2S	broad-alcohol	CO, 100 ppm
9	W2W	sulph-chlor	H_2_S, 1 ppm
10	W3S	methane-aliph	CH_3_, 100 ppm
